# In Vitro Evaluation of the Effect of Oral Probiotic *Weissella cibaria* on the Formation of Multi-Species Oral Biofilms on Dental Implant Surfaces

**DOI:** 10.3390/microorganisms9122482

**Published:** 2021-11-30

**Authors:** Mi-Sun Kang, Geun-Yeong Park

**Affiliations:** R&D Center, OraPharm, Inc., Seoul 04782, Korea; gypark@orapharm.com

**Keywords:** oral probiotics, titanium disc, implants, peri-implant mucositis, biofilm, qPCR, microscopic

## Abstract

Oral probiotics are beneficial bacteria that can help prevent periodontal disease. However, little is known about the effects of oral probiotics on the formation of implant biofilms. This study aimed to evaluate the effects of oral probiotics *Weissella cibaria* CMU and CMS1 in an in vitro complex biofilm model on titanium implant surfaces. First, it was identified through colony biofilm assay that *W. cibaria* CMU and CMS1 inhibit the formation of multi-species biofilms formed by eight types of bacteria. Two types of saliva-coated titanium discs inoculated with early (*Streptococcus gordonii, Streptococcus oralis, Streptococcus sanguinis, Actinomyces naeslundii,* and *Veillonella parvula*), secondary (*Fusobacterium nucleatum* and *Prevotella intermedia*), and late (*Porphyromonas gingivalis*) colonizers were treated with the oral probiotics and then incubated anaerobically for three days. The effects of oral probiotics on titanium disc biofilm formation were analyzed using culture methods, quantitative polymerase chain reaction (qPCR), and microscopic analysis. Both probiotics significantly inhibited the formation of biofilm, and all eight bacterial species were significantly reduced. The effectiveness of both probiotic strains was confirmed by all the methods used. Oral probiotics may have dramatically reduced the biofilm formation of secondary colonizers that act as bridges, thus inhibiting biofilm formation on the titanium surface. Our results suggest that the probiotic *W. cibaria* offers new possibilities for the prevention of peri-implant mucositis.

## 1. Introduction

When teeth are lost, implants are planted to manage the aesthetics and function of the natural teeth [1]. Titanium is the most commercially available dental implant material owing to its mechanical properties and biocompatibility [2,3]. However, the success rate of titanium transplantation into human bone decreases because of its low bone-bonding ability [4,5]. For successful implantation and maintenance, research into improving the bone response through changes in the surface area and the shape of various titanium surface treatments, such as machining, sandblasting with large grit and acid etching (SLA), and resorbable blasted media, has been continuously conducted [6,7,8]. This titanium surface treatment obviously improves the osseointegration of implants; however, it promotes the development of complex biofilms, making it difficult to maintain cleanliness [2,6,9].

Management is very important in order to use the implant well for more than 10 years after implantation. If plaque accumulates between the implant and the gums, bacteria can penetrate and cause inflammation. Dental implants are placed after the periodontal ligament nerve is removed. Therefore, implants are difficult to manage properly because there are no nerves that transmit pain from inflammation. The formation of complex biofilms on the exposed implant surface is a key factor in the pathogenesis of peri-implant diseases. In order to prevent disease development, it is necessary to understand the process of complex bacterial biofilm formation on the implant surface and identify functional substances that can inhibit biofilm formation.

Peri-implant mucositis is defined as peri-implant mucosal inflammation without continuous marginal peri-implant bone loss [10], while peri-implantitis is a pathological condition that occurs in the peri-implant tissue characterized by inflammation of the peri-implant connective tissue and progressive loss of supporting bone [11].

Antibacterial agents, such as chlorhexidine and cetylpyridinium chloride, and mechanical debridement are standard therapies of peri-implant mucositis and are secondary prevention measures for peri-implantitis [12]. However, since antibacterial agents only show temporary effects and may have side effects when used for a long period of time, a preventive agent that can be used on a daily basis is needed.

In recent years, oral probiotics have been shown to be beneficial in preventing or treating oral diseases, such as tooth decay, gingivitis, and periodontitis, by improving the microenvironment of the microbiota in the oral cavity [13]. Probiotics are defined as “living microorganisms that, when administered in appropriate amounts, confer a health benefit on the host” [14]. *Weissella cibaria*, a Gram-positive lactic acid bacterium, was first classified in 2002 [15], and it was identified as a dominant species of fermented foods such as kimchi and showed probiotic potential by inhabiting the oral cavity [16].

*W. cibaria* CMU improved oral health by inhibiting the proliferation of several oral bacteria and improving the bleeding index [17]. On the other hand, *W. cibaria* CMS1 lowered the plaque index by inhibiting the formation of *Streptococcus mutans* biofilm [18]. Both strains were confirmed to be safe as probiotics for oral care to prevent oral diseases [19].

Complex biofilm models in vitro have been developed and validated through biofilm models using early, secondary, and late colonizer bacteria [20,21,22]. Therefore, in this study, we aimed to determine whether the *W. cibaria* strains affect the in vitro multi-species biofilm formation on machined or SLA-treated titanium discs using various assays.

## 2. Materials and Methods

### 2.1. Bacterial Strains and Growth Conditions

Eight standard reference strains of *Actinomyces naeslundii* KCTC 5525, *Streptococcus gordonii* KCTC 5640, *Streptococcus oralis* ATCC 35307, *Streptococcus sanguinis* ATCC 10556, *Veillonella parvula* KCTC 5487, *Fusobacterium nucleatum* KCTC 2488, *Prevotella intermedia* ATCC 25611, and *Porphyromonas gingivalis* ATCC 33277 were used. All reference strains were grown on TSHMB agar plates under anaerobic conditions (AnaeroPack-Anaero; Mitsubishi Gas Chemical Co., Tokyo, Japan) at 37 °C for 3 d. TSHMB contains tryptic soy agar (MB cell, Kisan Bio, Seoul, Korea), supplemented with 5 μg/mL hemin (MB cell), 0.5 μg/mL menadione (MB cell), and 5% (*v*/*v*) sterile defibrinated sheep blood (MB cell). *W. cibaria* CMU (oraCMU^®^; OraPharm, Inc., Seoul, Korea) and *W. cibaria* CMS1 (oraCMS1^®^; OraPharm), used as oral care probiotics, were grown aerobically in Mann, Rogosa, and Sharpe (MRS) broth (Difco, Detroit, MI, USA) at 37 °C for 16 h. To prepare the cell-free supernatants (CFS) of bacteria, cells were removed using centrifugation (4000× *g*, 20 min, 4 °C), and the CFS was filtrated (0.22 μm; JET BIOFIL, Guangzhou, China).

### 2.2. Saliva Preparation

Unstimulated saliva from healthy volunteers was obtained in 10 mL aliquots at least 1.5 h after eating, drinking, or brushing teeth. To reduce salivary protein aggregation, each saliva sample was treated with 2.5 mM DL-dithiothreitol (Sigma, St. Louis, MO, USA) with continuous stirring for 10 min. After centrifugation (9000× *g*, 10 min, 4 °C), the resulting supernatant was diluted (1:1) with phosphate buffered saline (PBS), filtered through a 0.22 μm pore size filter, and stored at −20 °C until the experiment. Saliva samples were inoculated on TSHMB agar plate and cultured under anaerobic conditions at 37 °C for 3 d to confirm that there was no bacterial growth.

### 2.3. Formation of Multi-Species Colony Biofilm

To investigate the formation of multi-species colony biofilms over time, colony biofilm assays were performed as described by Kang et al. [23] with slight modifications. Briefly, sterile polycarbonate semipermeable membrane filters (diameter, 25 mm; pore size, 0.2 μm; Merck Millipore, Dublin, Ireland) were placed on TSHMB agar overnight to permeate the medium and allow growth of surface biofilms. Colonies of eight bacterial species were diluted to obtain an optical density of 0.5 at 600 nm (OD_600_) (1 × 10^7^ colony forming units (CFU)/mL), mixed in equal volumes, spotted onto the center of individual membrane filters (100 μL), and incubated anaerobically at 37 °C. The membrane-supported complex biofilm was measured after 1, 3, and 6 d using the viable cell counting method and quantitative polymerase chain reaction (qPCR).

### 2.4. Evaluation of W. cibaria on Multi-Species Colony Biofilm Formation

The effects of *W. cibaria* CMU and CMS1 on multi-species oral biofilms in vitro were determined using the colony biofilm assay as described in Section 2.3. After incubation for 1 d, the membrane-supported complex biofilm was transferred to fresh TSHMB agar plates (0.8% soft agar), inoculated with CFS of *W. cibaria* CMU or CMS1 in 1:1 ratio, and incubated anaerobically at 37 °C for 5 d. TSHMB agar plates (0.8% soft agar) mixed 1:1 with MRS were used as negative control. The effects of *W. cibaria* on colony biofilm formation were analyzed using culture methods, qPCR, and microscopic analysis.

### 2.5. Evaluation of W. cibaria on the Formation of Multi-Species Oral Biofilms on Dental Implant Surface

Sterile discs (diameter, 8 mm; thickness, 2 mm) made of two different surface materials (Polybiotech, Gwangju, Korea) were used: (1) titanium with a machined grade 2 surface (Ti-M) and (2) titanium with an SLA grade 2 surface (Ti-SLA). A total of 0.2 mL saliva was dropped onto the discs and then incubated with shaking (200 rpm) at 37 °C for 4 h before use. Biofilms were grown using the method described by Sánchez et al. [20] with some modifications. Briefly, the colonies of eight bacterial strains cultured on TSHMB agar were suspended in TSHMB broth using a sterilized loop, and the OD_600_ was adjusted to 0.5. Each bacterium was mixed in equal amounts, and 0.5 mL was added to the 24-well plates (SPL Life Science, Pocheon, Korea) containing the sterile discs. Then, 0.5 mL *W. cibaria* CMU or CMS1 CFS was inoculated on each well, and the plates were incubated anaerobically for 3 d. Plates that were not inoculated with CFS of either probiotics were used as positive control. The effects of *W. cibaria* on biofilm formation on titanium discs were analyzed using culture methods, qPCR, and microscopic analysis.

### 2.6. Measurement of Viable Cells and Quantity of Biofilm

Colony biofilms were harvested after 0 h, 1, 3, and 6 d, homogenized in an ice bath at high speed for 40 s (Tissue Ruptor II; QIAGEN, Hilden, Germany) in 10 mL of peptone water (MB cell) previously reduced anaerobically, serially diluted in peptone water, and plated in triplicates to count viable CFU. The antibacterial activities of *W. cibaria* CMU and CMS1 were determined using phase-contrast microscopy (DCS 6002; Dr. PREVENT, Seoul, Korea). To measure the bacterial viability of the disc biofilm, the discs cultured for 3 d were washed three times with PBS, transferred to a tube containing peptone water, and vortexed vigorously for 2 min. After serial dilution and inoculation on TSHMB agar, the plates were incubated anaerobically at 37 °C for 5 d. The biofilm was quantified using a microplate reader (VersaMax, Molecular Devices, San Jose, CA, USA) at 600 nm. Then, the relative amount of disc biofilm was also measured spectrophotometrically. Briefly, the culture medium was removed, and the disc was washed three times with sterile distilled water (DW) then air-dried for 5 min. Each disc in the well was stained with 0.1% crystal violet for 15 min, and the discs were washed three times with sterile DW. Five hundred microliters of absolute ethanol was added to the disc in each well to dissolve the stain absorbed by the biofilm. The dissolved stain (200 μL) was dispensed into 96-well plates (SPL Life Science), and the absorbance was measured at 595 nm using a microplate reader. The relative quantitative value for the *W. cibaria*-treated groups was calculated by setting the control biofilm concentration to 1.

### 2.7. qPCR

To quantify the number of eight bacterial species in the disc biofilm using qPCR, the homogenized suspension obtained as described above was centrifuged (5000× *g*, 10 min, 4 °C), and the pellet was washed with PBS. Then, genomic DNA was extracted from the pellet using a QIAamp DNA Mini Kit (QIAGEN) following the manufacturer’s instructions. The standard curves for qPCR were prepared using 10-fold serial dilutions of DNA from 10^2^ to 10^8^. The cycle threshold started from the logarithmic phase of a known DNA concentration, and the number of bacteria per sample was determined using the standard curve. Primers that target species-specific genes for 16S rRNA were synthesized by Macrogen (Seoul, Korea) (Table 1). For qPCR, 3 μL of DNA template, 5 μL of 2X SYBR Green PCR Master Mix (Applied Biosystems, Thermo Fisher Scientific, Waltham, MA, USA), and 1 μL of each primer (10 pmoL) were mixed to make a final volume of 10 μL. The qPCR was performed using the Rotor-Gene Q system (QIAGEN), and the reaction conditions were as follows: initial denaturation at 95 °C for 2 min, followed by 40 cycles at 95 °C for 15 s and 60 °C for 1 min. The results were analyzed using the Rotor-Gene Q series software 2.3.1.

### 2.8. Confocal Laser Scanning Microscopy (CLSM) Analysis

Colony biofilms were gently washed with sterile DW and stained with a Filmtracer LIVE/DEAD BacLight bacterial viability kit (Invitrogen, Thermo Fisher Scientific, Waltham, MA, USA) to visualize viable (in green, Syto9) and dead cells (in red, propidium iodide). Staining was performed according to the manufacturer’s instructions. Membrane-supported colony biofilms were mounted onto glass slides with BacLight mounting oil (Invitrogen) and analyzed using CLSM (Leica Microsystems, Wetzlar, Germany). To perform CLSM analysis on titanium disc biofilm formation, the stained discs were inverted and placed on a confocal dish (SPL Life Science) equipped with mounting oil for analysis. The stained biofilm was imaged at 800× magnification, and the live/dead cell ratio was calculated using the Las X software package (Leica Microsystems).

### 2.9. Scanning Electron Microscopic (SEM) Analysis

SEM analysis was performed as described by Ceresa et al. [26], with minor modifications. Briefly, titanium discs with surface biofilm were washed three times with PBS and fixed in glutaraldehyde solution (2.5% *w*/*v*, Sigma) for 2 h at 4 °C. Then, the glutaraldehyde solution was removed, and the specimen was washed three times with PBS for 5 min. Osmium tetroxide solution (1% *w*/*v*, Sigma) was added to the samples for 1 h at 25 ℃ and rinsed twice (PBS and DW each) for 5 min. After fixation, the titanium discs with biofilms were dehydrated in a graded ethanol series (50%, 60%, 70%, 80%, 90%, and 100%) for 10 min each. For substitution, hexamethyldisilazane (Sigma) was added twice for 10 min. After drying overnight, samples were sputter-coated with gold and observed using SEM (Hitachi, Model: SU-70, Tokyo, Japan) at an accelerating voltage of 15.0 kV.

### 2.10. Statistical Analysis

Data are presented as the mean ± standard deviation of triplicate measurements. Statistical analyses were performed using SPSS Statistics version 21.0 for Windows (IBM Corp., Armonk, NY, USA). The Mann–Whitney *U* test was used to identify any statistically significant differences between the control and experimental groups (*p* < 0.05).

## 3. Results

### 3.1. Formation of Multi-Species Colony Biofilm

**Figure 1:**
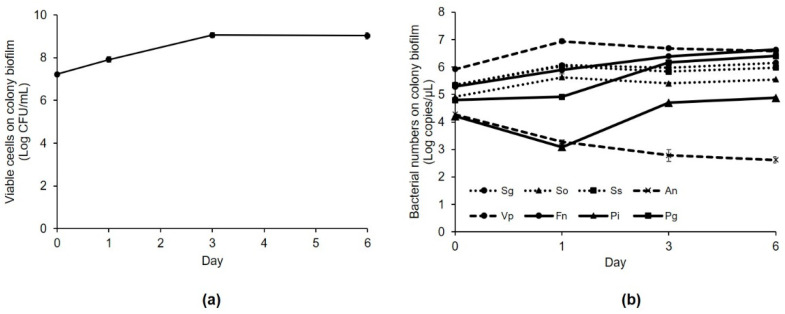
Multi-species colony biofilm formation. (**a**) Number of viable cells in the colony biofilm over time. (**b**) Change in the number of bacteria in the colony biofilm over time measured using quantitative polymerase chain reaction. Sg, *S. gordonii*; So, *S. oralis*; Ss, *S. sanguinis*; An, *A. naeslundii*; Vp, *V. parvula*.; Fn, *F. nucleatum*; Pi, *P. intermedia*; Pg, *P. gingivalis*.

The formation of multi-species colony biofilm was measured using the viable cell count method. The number of viable cells increased after incubation for 6 days, but the values on day 3 and 6 were similar (Figure 1a). The qPCR was also performed to quantify the changes in the number of the eight bacterial species during colony biofilm formation for 6 d. The results showed that the numbers of *F. nucleatum, P. intermedia,* and *P. gingivalis* increased with the greatest variation (Figure 1b).

### 3.2. Effects of W. cibaria on Multi-Species Colony Biofilm Formation

**Figure 2:**
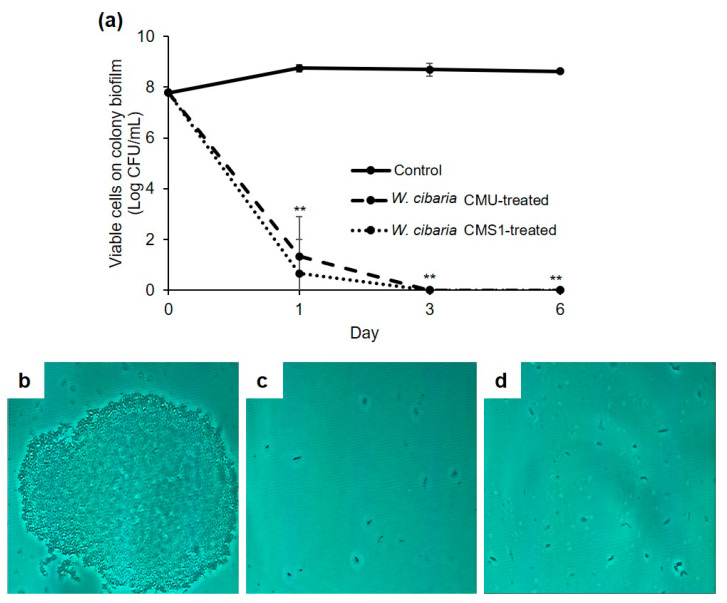
Effects of *W. cibaria* CMU and CMS1 on multi-species biofilm during co-culture at 1, 3, and 6 d measured using (**a**) viable cell counting and (**b**–**d**) phase-contrast microscopic analysis. (**a**) Number of viable cells in the colony biofilm over time. Significant differences as compared to control were determined as ** *p* ≤ 0.001. (**b**) Control, (**c**) *W. cibaria* CMU-treated, and (**d**) *W. cibaria* CMS1-treated observed on the sixth day. Magnification, 4000×.

**Figure 3:**
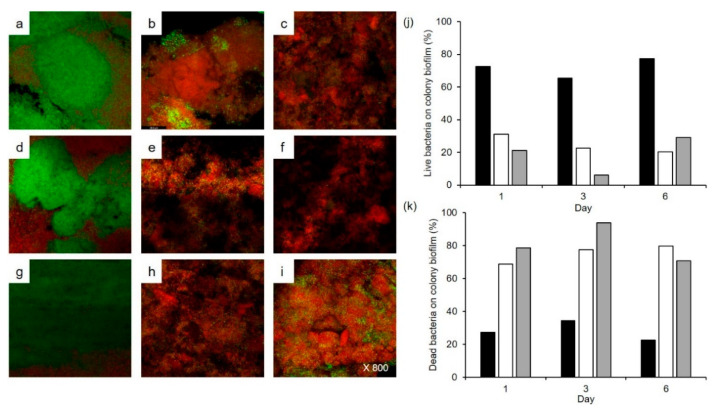
Effects of *W. cibaria* CMU and CMS1 on multi-species biofilm during co-culture at 1, 3, and 6 d observed using confocal laser scanning microscopy to visualize live/dead bacteria. (**a**–**c**) Control, *W. cibaria* CMU-treated, and CMS1-treated for 1 d, respectively; (**d**–**f**) control, *W. cibaria* CMU-treated, and *W. cibaria* CMS1-treated for 3 d, respectively; (**g**–**i**) treated control, *W. cibaria* CMU-treated, and *W. cibaria* CMS1-treated for 6 d, respectively. Magnification, 800×. (**j**) Percent live bacteria on colony biofilm; and (**k**) percent dead bacteria on colony biofilm. Black bar, control; white bar, *W. cibaria* CMU-treated; grey bar, *W. cibaria* CMS1-treated.

The inhibitory effects of *W. cibaria* strains on colony biofilm formation were evaluated using three methods. The number of viable cells in the colony biofilm of the control group was 8.62 log CFU/mL on the sixth day, whereas that in the *W. cibaria* CMU and CMS1-treated groups significantly decreased by more than 7 logs from the first day, and no viable cells were observed from the third day (*p* ≤ 0.001) (Figure 2a). In phase-contrast microscopy, numerous bacterial cells were observed in the control group, whereas only a very small number of cells were observed in the *W. cibaria* CMU-and CMS1-treated groups (Figure 2b–d). As shown in Figure 3, the live/dead bacteria ratio in the *W. cibaria* CMU or CMS1-treated group and the control group over time was also evident in the CLSM and LIVE/DEAD staining results.

### 3.3. Effects of W. Cibaria on the Formation of Multi-Species Oral Biofilms on Dental Implant Surface

#### 3.3.1. Measurement of Viable Cells and Quantity of Implant Biofilms

**Figure 4:**
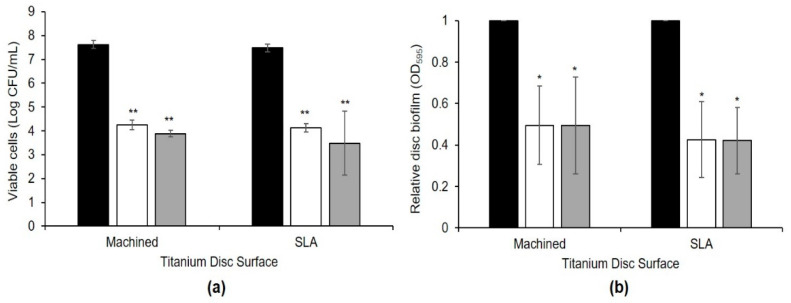
Inhibitory effects of *W. cibaria* CMU and CMS1 on multi-species implant biofilm formation measured using (**a**) viable cell counting and (**b**) relative biofilm amount. Titanium discs with machined or sandblasted with large grit and acid etching (SLA) surface were used. (**a**) Number of viable cells in the biofilm formed on the discs for 3 d; (**b**) stained biofilm formed for 3 d using crystal violet and measured at OD_595_. Black bar, control; white bar, *W. cibaria* CMU-treated; grey bar, *W. cibaria* CMS1-treated. Significant differences as compared to control determined as * *p* < 0.05, ** *p* ≤ 0.001.

**Figure 5:**
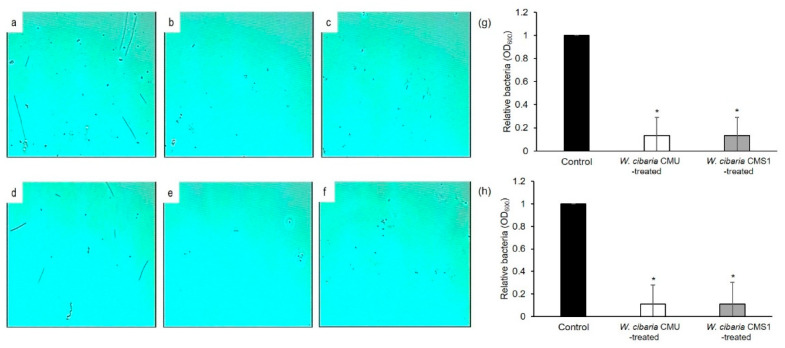
(**a**–**f**) Phase-contrast microscopic analysis and (**g**,**h**) absorbance at 600 nm of the inhibitory effects of *W. cibaria* CMU and CMS1 on the formation of multi-species implant biofilms using (**a**–**g**) titanium discs with machined or (**d**–**h**) SLA surface. (**a**,**d**) Control; (**b**,**e**) *W. cibaria* CMU-treated; (**c**,**f**) *W. cibaria* CMS1-treated. Magnification, 4000×. (**g**) Relative quantity of biofilms on titanium discs with machined surface; (**h**) relative quantity of biofilms on titanium discs with SLA surface. Black bar, control; white bar, *W. cibaria* CMU-treated; grey bar, *W. cibaria* CMS1-treated. Significant differences as compared to control determined as * *p* < 0.05.

The bacterial viability on the disc biofilm cultured for 3 d was measured. Results showed that the number of viable cells in *W. cibaria* CMU- and CMS1-treated Ti-M and Ti-SLA were significantly lower than that of the control group (*p* ≤ 0.001) (Figure 4a). Statistically significant decreases (*p* < 0.05) in the relative quantity of disc biofilms measured at 595 nm in the *W. cibaria*-treated group were observed as compared to the control group (Figure 4b). In addition, as shown in Figure 5, statistically significant decreases (*p* < 0.05) in the absorbance value at 600 nm and the quantity of disc biofilms measured using a phase-contrast microscope in the *W. cibaria*-treated group were observed as compared to the control group.

#### 3.3.2. Quantitative Analysis of Implant Biofilms Using qPCR

**Figure 6:**
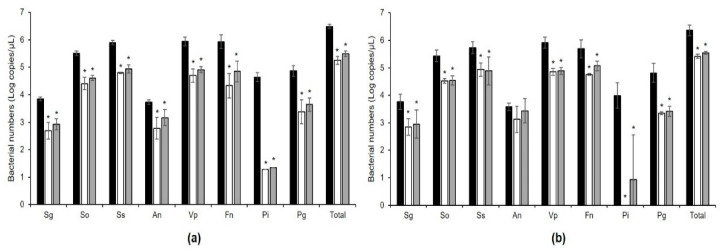
qPCR analysis of the inhibitory effects of *W. cibaria* CMU and CMS1 on the formation of multi-species implant biofilms using the titanium discs with (**a**) machined or (**b**) SLA surface. Sg, *S. gordonii*; So, *S. oralis*; Ss, *S. sanguinis*; An, *A. naeslundii*; Vp, *V. parvula*; Fn, *F. nucleatum*; Pi, *P. intermedia*; Pg, *P. gingivalis*. Total, total bacteria. Black bar, control; white bar, *W. cibaria* CMU-treated; grey bar, *W. cibaria* CMS1-treated. Significant differences as compared to control determined as * *p* < 0.05.

Figure 6 shows the qPCR quantification of eight bacterial species that formed biofilms on Ti-M and Ti-SLA surfaces when treated with *W. cibaria* CMU or CMS1 for 3 d. Eight types of bacteria were detected in both discs, and a statistically significant reduction (*p* < 0.05) in most of the bacteria were observed in *W. cibaria*-treated groups compared to the control group.

#### 3.3.3. CLSM Analysis of Implant Biofilms

**Figure 7:**
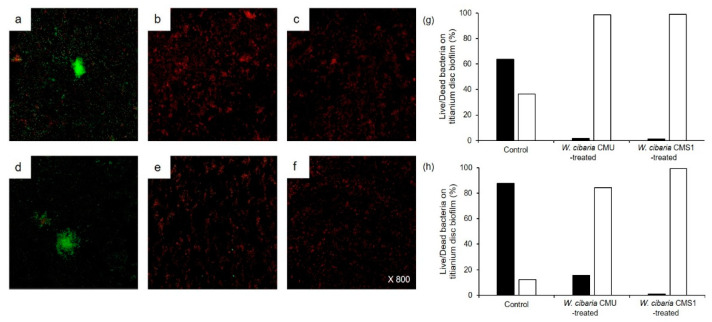
CLSM analysis of multi-species implant biofilms on titanium discs with (**a**–**g**) machined or (**d**–**h**) SLA surface. (**a**,**d**) Control; (**b**,**e**) *W. cibaria* CMU-treated; (**c**,**f**) *W. cibaria* CMS1-treated. Magnification, 800×. (**g**) Live/dead cell ratios on titanium discs with machined surface; (**h**) live/dead cell ratios on titanium discs with SLA surface. Black bar, live bacteria; white bar, dead bacteria.

CLSM analysis was used to observe the inhibitory effects of *W. cibaria* strains on multi-species implant biofilm formation. In both titanium discs, live bacteria in the control group were observed in the biofilm, whereas dead bacteria were mostly found in the group treated with *W. cibaria* strains (Figure 7).

#### 3.3.4. SEM Analysis of Implant Biofilms

**Figure 8:**
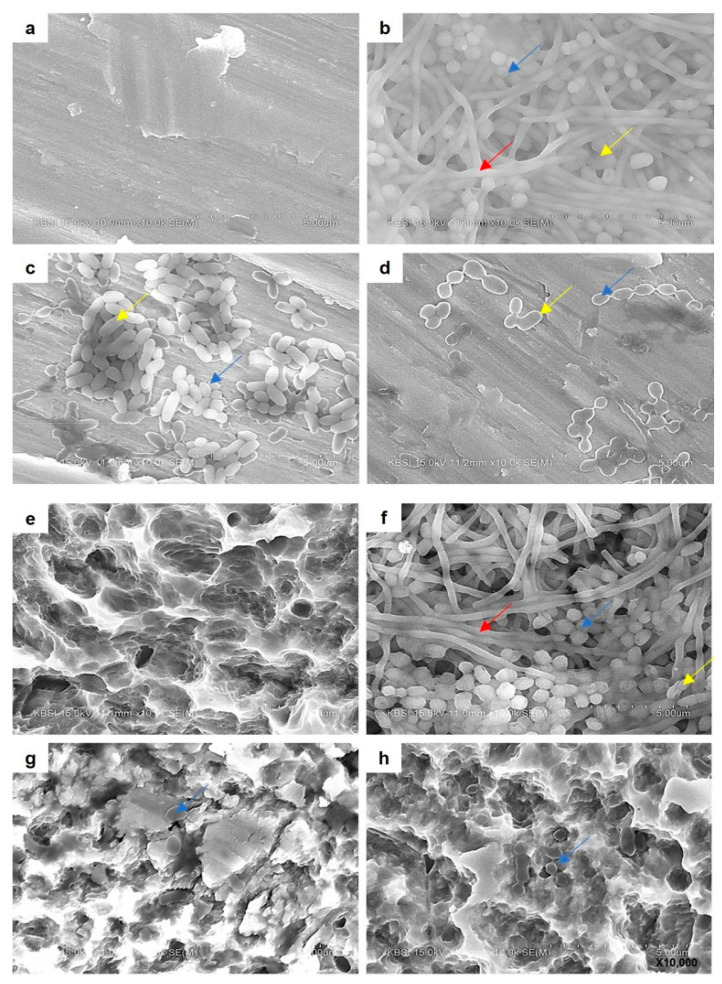
Images obtained using scanning electron microscope of multi-species implant biofilms on titanium discs with (**a**–**d**) machined or (**e**–**h**) SLA surface. (**a**,**e**) Untreated surface; (**b**,**f**) control; (**c**,**g**) *W. cibaria* CMU-treated; (**d**,**h**) *W. cibaria* CMS1-treated. Red arrows, spindle-shaped rods; blue arrows, cocci; yellow arrows, rods. Magnification, 10,000×.

SEM analysis was also performed to observe the inhibitory effects of *W. cibaria* strains on multi-species implant biofilm formation (Figure 8). After 3 d of incubation, bacterial biofilms covered the implant surface, and spindle-shaped rods and streptococcal bacteria were evidently recognized in the control group (Figure 8b,f). However, spindle-shaped and rod-shaped bacteria were not observed in the *W. cibaria* CMU- (Figure 8c,g) and CMS1-treated (Figure 8d,h) groups, and only a very small number of short streptococci and rod bacteria were observed.

## 4. Discussion

*W. cibaria* is a short, rod-shaped, Gram-positive, non-spore-forming, non-motile, heterofermentative, and catalase-negative lactic acid bacterium [15]. Strains *W. cibaria* CMU and CMS1 were isolated from saliva samples of children with little supragingival plaque and no oral diseases, including dental caries [18]. Do et al. [16] reported that *W. cibaria* CMU inhibited the proliferation of periodontal pathogens *F. nucleatum, P. gingivalis, P. intermedia*, and *T. forsythia* in beagles.

Biofilm formations have been steadily conducted using implant materials [2,3,4], but there have been few studies on the effects of oral probiotics on implant biofilm formation. To the best of our knowledge, there are no in vitro studies evaluating the efficacy of the *W. cibaria* strains on implant biofilm formation.

When the dental implant is inserted into the oral cavity, the sterile implant surface is exposed to microorganisms and forms salivary-derived pellicles on the oral mucosa, which results in the formation of oral bacterial biofilms. These biofilms induce an immune inflammatory response in the peri-implant tissues due to quantitative and qualitative changes in bacterial composition and virulence, eventually leading to peri-implant disease [2].

Peri-implant infections are one of the most common causes of implant failure [27]. For efficacy studies on peri-implant mucositis, Butera et al. [28] proposed a probiotic alternative to chlorhexidine at the periodontal and implant level to reduce the incidence of *F. nucleatum* and *P. intermedia*. They also expanded the study and reported that natural substances such as ozone water reduce peri-implant mucositis [29]. The study by Ciandrini et al. [30] showed that live, heat-killed, and CFS of *Lactobacillus* sp. inhibited *S. mutans* and *S. oralis* biofilm formation on titanium discs. It has also been suggested that *Latobacillus plantarum* lipoteichoic acid is a potential anti-biofilm agent for the treatment or prevention of apical periodontitis, which is mainly caused by multispecies biofilms such as *A. naeslundii*, *Lactobacillus salivarius*, *S. mutans,* and *Enterococcus faecalis*. This inhibitory effect was confirmed by crystal violet assay and CLSM and SEM analysis, but did not show the quantitative analysis of the pathogens as in our study [31]. In addition, the current study is differentiated from other studies in that probiotics inhibited the formation of eight types of multispecies biofilms on titanium discs.

Because of the clinical implications of biofilms on implant surfaces, there have been studies on in vitro biofilm models of oral microbes [9]. In these models, *A. naeslundii* and *S. gordonii* have been mainly used as early colonizers on the tooth surface, *V. parvula* as a secondary colonizer, *F. nucleatum* as an intermediate colonizer, and *P. gingivalis* as a late colonizer [9]. In the present study, in addition to these bacteria, *S. oralis* and *S. sanguinis* were used as initial colonizers, and *P. intermedia* was used as an intermediate colonizer.

Furthermore, in this study, before observing the effect of probiotics on biofilm formation on the implant surface, colony biofilm formation was observed in order to examine the process of oral microorganisms forming multi-species biofilms and the effect of *W. cibaria* on multi-species biofilm formation. Among the eight bacterial species used in this study, the intermediate and late colonizers, *F. nucleatum*, *P. intermedia*, and *P. gingivalis*, increased rapidly on the third day of incubation, and the initial colonizers showed a tendency to decrease. This result is consistent with the fact that biofilms accumulate on the tooth surface in vivo, and the supragingival and subgingival biofilms along the gingival margin allow for mostly anaerobic bacterial communities, which can cause periodontal disease [32].

Titanium, used as an implant material, has excellent mechanical properties, such as a relatively low modulus of elasticity and high corrosion resistance, and is widely used as a biomedical metal material [4,5] because of its good biocompatibility. Implants with mechanical and chemical surface treatments have been commercialized to enhance bone bonding ability [6,7,8].

In the present study, biofilm formations on machined and SLA-treated titanium discs were compared. *W. cibaria* CMU and CMS1 showed similar inhibition of biofilm formation on the titanium surface regardless of the surface type. Changes in eight bacterial strains were observed using qPCR, and when *W. cibaria* CMU and CMS1 were treated, there was a statistically significant decrease in not only the eight types of microorganisms but also in the total bacteria. In particular, a marked decrease in the abundance of *P. intermedia* was observed. SEM analysis also showed various microbial communities in the control group, but only a small number of microbial morphologies were observed in the *W. cibaria* CMU- and CMS1-treated groups. These results suggest that probiotics prevent late colonization of bacteria, such as *P. gingivalis*, by reducing intermediate colonizers, such as *F. nucleatum* and *P. intermedia*. This study supports previous reports that the use of probiotics and their metabolites is a promising approach by which to reduce the impact of biofilms in medical devices [30,31].

## 5. Conclusions

This study is the first to confirm the effect of oral probiotic *W. cibaria* strains CMU and CMS1 on the formation of multi-species oral biofilm in vitro and in a titanium disc using various analytical methods. This study suggests that oral probiotics can be used as an adjunct therapy to prevent peri-implant infections by inhibiting the formation of implant biofilms.

## Figures and Tables

**Table 1:** Primer sequences used for the quantification of the target bacterial species in the biofilm.

Bacteria	Sequence (5′–3′)	Length (bp)	References
*S. gordonii*			
Forward	CCGTCACACCACGAGAGTTT	104	This study
Reverse	CCTTGTTACGACTTCACCCCA		
*S. oralis*			
Forward	CAACGATACATAGCCGACCTGAG	102	[21]
Reverse	TCCATTGCCGAAGATTCC		
*S. sanguinis*			
Forward	GATCCTGGCTCAGGACGAAC	103	This study
Reverse	TACTCACCCGTTCGCAACTC		
*A. naeslundii*			
Forward	GGCTGCGATACCGTGAGG	104	[21]
Reverse	TCTGCGATTACTAGCGACTCC		
*V. parvula*			
Forward	CCGTGATGGGATGGAAACTGC	106	[24]
Reverse	CCTTCGCCACTGGTGTTCTTC		
*F. nucleatum*			
Forward	TCGTGTCGTGAGATGTTGGG	156	This study
Reverse	GTAGCCCAGCGTATAAGGGG		
*P. intermedia*			
Forward	TTGGGGAGTAAAGCGGGCA	151	This study
Reverse	CGCTTAACAGACCGCCTACA		
*P. gingivalis*			
Forward	AGGCAGCTTGCCATACTGCG	100	This study
Reverse	ACTGTTAGCAACTACCGATGT		
Total bacteria			
Forward	CCATGAAGTCGGAATCGCTAGT	86	[25]
Reverse	GCTTGACGGGCGTGTG		

## Data Availability

The data presented in this study are available upon request from the corresponding author.
